# Case Report: Idiopathic Wunderlich syndrome complicated by a perinephric abscess with fistula formation to the descending colon

**DOI:** 10.3389/fmed.2026.1575103

**Published:** 2026-02-04

**Authors:** Tae Won Lee, Eunjin Bae, Ha Nee Jang, Sehyun Jung, Seunghye Lee, Se-Ho Chang, Dong Jun Park

**Affiliations:** 1 Department of Internal Medicine, Gyeongsang National University Changwon Hospital, Changwon, Republic of Korea; 2 Department of Internal Medicine, Gyeongsang National University College of Medicine, Jinju, Republic of Korea; 3 Institute of Health Science, Gyeongsang National University, Jinju, Republic of Korea; 4 Department of Internal Medicine, Gyeongsang National University Hospital, Jinju, Republic of Korea

**Keywords:** colon fistula, kidney rupture, perinephric abscess, subcapsular hematoma, Wunderlich syndrome

## Abstract

Wunderlich syndrome (WS) is a rare clinical condition characterized by spontaneous, non-traumatic renal hemorrhage. Although secondary infection of a renal hematoma has occasionally been reported, progression to a perinephric abscess with fistula formation to the descending colon has not been previously documented. We present a unique case of idiopathic WS in a 61-year-old diabetic woman with poorly controlled blood sugar who developed a spontaneous left subcapsular hematoma without an identifiable etiology. During hospitalization, follow-up contrast-enhanced computed tomography (CECT) revealed interval enlargement of the hematoma with new peripheral enhancement, consistent with abscess formation. Percutaneous catheter drainage confirmed secondary infection. Despite initial clinical stabilization, subsequent computed tomography (CT) demonstrated intralesional gas and progressive inflammatory changes involving the adjacent descending colon. A catheter tubogram confirmed a fistulous connection between the abscess cavity and the descending colon. The patient underwent fecal diversion with ileostomy, in addition to targeted antimicrobial therapy and image-guided drainage. Serial imaging over the following weeks demonstrated progressive resolution of both the abscess cavity and fistula tract, allowing catheter removal and discharge in a stable condition 70 days after hospitalization. This case highlights that even idiopathic WS may evolve into severe infectious complications, particularly in individuals with risk factors such as long-standing diabetes mellitus with suboptimal glucose control. Physicians should be aware that WS can be complicated by secondary bacterial infection, leading to abscess formation and fistula development between the abscess and the colon. Prompt and accurate diagnosis, along with appropriate management, is essential for resolving these complications.

## Introduction

Wunderlich syndrome (WS) is a rare clinical syndrome characterized by the acute onset of a spontaneous, non-traumatic renal hemorrhage into the subcapsular, perinephric, and/or pararenal spaces (1, 2). It is classically characterized by Lenk’s triad, which includes acute flank pain, flank mass, and hypovolemic shock. However, patients may present with various symptoms ranging from non-specific flank or abdominal pain to serious clinical conditions such as hypovolemic shock, requiring therapeutic intervention or emergency radical surgery (3, 4). The etiological factors of WS include renal neoplasms, renal vascular diseases, renal infections, nephritis, kidney failure, hematologic and coagulation abnormalities, and renal cystic disorders. In approximately 5–10% of patients with WS, no renal or systemic abnormality is identified on imaging (4).

A perinephric abscess is defined as purulent material that accumulates in the perinephric space and accounts for 0.02% of all abdominal abscesses (5). It frequently occurs because of parenchymal disruption subsequent to fulminating pyelonephritis, particularly as a consequence of an obstructing infectious stone (6). Less commonly, the source of infection is the renal carbuncle or renal abscess that ruptures into the perinephric space. The organism in these examples is *Staphylococcus aureus*, which classically metastasizes from skin infections or needle injections. Some cases of perinephric abscess complicated by traumatic renal biopsy have been reported (7). In this case, it is usually caused by a secondary bacterial infection of a perinephric hematoma. To the best of our knowledge, no previous reports have described WS complicated by a secondary bacterial infection leading to a perinephric abscess and subsequent fistula formation to the descending colon. This case highlights a rare but clinically significant complication pathway that has not been documented in the previous literature.

## Case report

A 61-year-old Korean woman was referred to our hospital with persistent left flank pain that started 9 days prior to admission. She initially visited the primary clinic and took medication for irritable bowel syndrome for 7 days. However, she had persistent flank pain; therefore, she visited a secondary hospital, where she was admitted and underwent blood and urine tests and a computed tomography (CT) scan. She was transferred to our hospital due to acute kidney injury (AKI) and a large spontaneous subcapsular hematoma on the left kidney. The patient had a 10-year history of diabetes, hypertension, and hyperlipidemia, and her medications included glimepiride 3 mg, metformin 1,000 mg, teneligliptin 20 mg, pravastatin 20 mg, and fimasartan 60 mg. She denied any genetic diseases, recent travel, or the use of other drugs, antiplatelet agents, or anticoagulants. She did not experience fever, constipation, or diarrhea. She had no urinary symptoms, such as frequency, dysuria, or urgency. She had no recent history of trauma.

Her vital signs were as follows: blood pressure (BP), 110/58 mmHg; body temperature (BT), 36.5 °C; heart rate (HR), 88 beats per min; and respiratory rate (RR), 18 breaths per min. She was alert and oriented, with no abnormalities noted during the neurological examination. Conjunctival pallor was present, but the sclerae were not icteric. No palpable cervical lymphadenopathy was observed. Lung auscultation revealed no wheezing or murmurs. There was no palpable organ in the abdomen, but there was tenderness on percussion over the left flank area. No bruise was detected in the abdomen or back area. No skin lesion was found anywhere on the body. There was no pitting edema in her lower extremities.

The initial laboratory findings are shown in Table 1. Clinically relevant abnormalities included leukocytosis (13.95 × 10^9^/L), elevated C-reactive protein levels (90.6 mg/L), and AKI with a serum creatinine of 3.28 mg/dL. Her HbA1c was 8.5%. Autoimmune workup, including ANA patterns, anti-dsDNA antibodies, ANCA, and anti-GBM antibodies, was negative. Urinalysis revealed a specific gravity of 1.015 ± protein, 2 + blood cells, and 2 + white blood cells. The urine albumin–creatinine ratio was 54.32 mcg/mg creatinine (<30 mcg/mg). No bacterial growth was observed in the initial urine or blood cultures. Atypical cells were not found in urine cytology.

**Table 1:** Baseline laboratory results at admission.

Parameters	Results	Reference range
Complete blood count (CBC)		
White blood cells (×10^9^/L)	13.95	4.0–10.0
Neutrophils (%)	87.4	—
Lymphocytes (%)	4.6	—
Monocytes (%)	7.9	—
Hemoglobin (g/dL)	10.9	12–16
Platelets (×10^9^/L)	363	130–400
Biochemical findings		
BUN (mg/dL)	27	9–23
Creatinine (mg/dL)	3.28	0.55–1.02
Total protein (g/dL)	6.4	5.7–8.2
Albumin (g/dL)	3.4	3.2–4.8
Total cholesterol (mg/dL)	85	120–199
ALP (IU/L)	122	30–120
AST (U/L)	11	1–33
ALT (U/L)	11	10–49
Glucose (mg/dL)	256	74–106
Sodium (mmol/L)	131	136–145
Potassium (mmol/L)	4.1	3.5–5.1
Chloride (mmol/L)	99	98–107
CRP (mg/L)	90.6	0–4.9
HbA1c (%)	8.5	4.2–5.9
C3 (mg/dL)	126.3	90–180
C4 (mg/dL)	35.9	10–40
Blood coagulation test		
Prothrombin time (sec)	13.6	9.4–12.5
aPTT (sec)	29.4	25.1–36.5
Autoantibody		
ANA	Positive (1:80)	Negative
Anti-dsDNA IgG/IgM	Negative	Negative
MPO/PR3 ANCA	Negative	Negative
Anti-GBM antibody	Negative	Negative

ALP, alkaline phosphatase; ALT, alanine aminotransferase; ANA, anti-nuclear antibody; ANCA, anti-neutrophilic cytoplasmic antibody; aPTT, activated partial thromboplastin time; AST, aspartate aminotransferase; BUN, blood urea nitrogen; CRP, C-reactive protein; GBM, glomerular basement membrane; MPO, myeloperoxidase.

**Figure 1:**
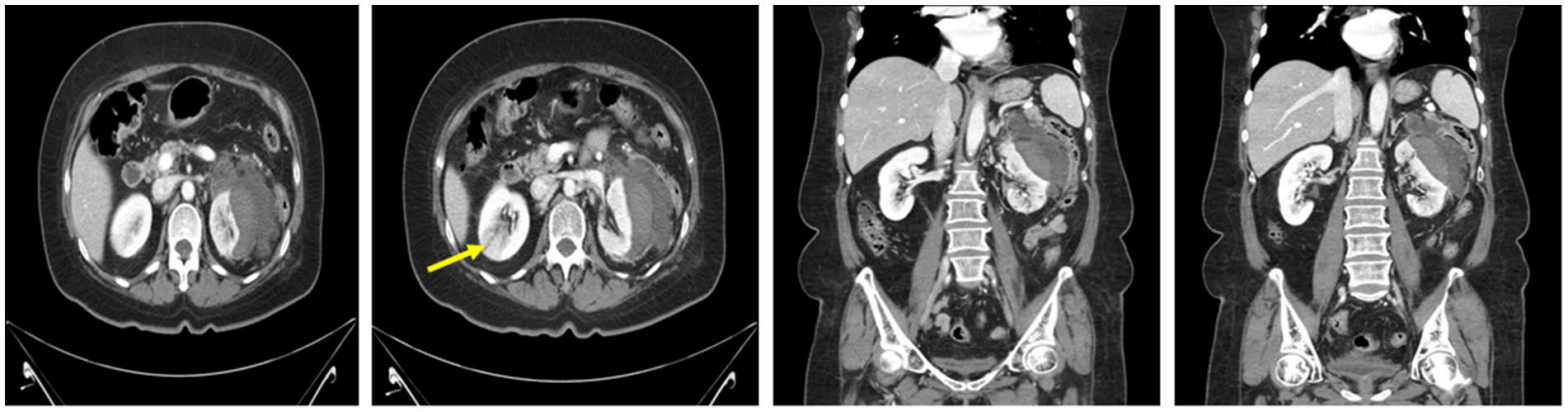
Contrast-enhanced CT showing a non-enhancing subcapsular hematoma measuring 8 cm in length on the left kidney, accompanied by fluid collection in the perinephric space and suspicious wedge-shaped heterogeneous areas of poor enhancement (arrow) representing acute pyelonephritis in the right kidney.

**Figure 2:**
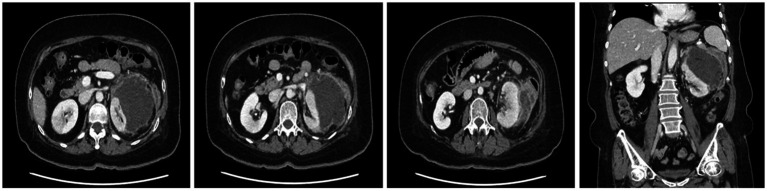
Follow-up CT showing an approximately 11 cm perinephric abscess with peripheral enhancement, medial displacement of the left kidney, and adjacent edematous changes in the descending colon.

A CT scan performed at the secondary hospital revealed an 8 cm subcapsular hematoma on the left kidney, without evident etiologies causing hemorrhage, and a wedge-shaped density in the right kidney, which was compatible with acute pyelonephritis (APN). No active renal hemorrhage was found on CT (Figure 1). Conservative management, including saline hydration and sugar, blood pressure, and pain control, was carried out, and intravenous ciprofloxacin was administered for APN management after admission. No fever was observed until the 10th day after admission, and vital signs were stable; however, the flank pain persisted. A CECT was performed on the 10th day of hospitalization after the confirmation of restored renal function (serum creatinine: 0.89 mg/dL). The CECT revealed an enlarged fluid collection with peripheral enhancement, suggestive of a perinephric abscess (Figure 2). A percutaneous drainage (PCD) procedure was performed, initially aspirating approximately 100 mL of reddish fluid, and the catheter was left in the hematoma for continuous drainage. *Klebsiella pneumoniae* grew in the drainage fluid culture and was highly sensitive to ciprofloxacin.

**Figure 3:**
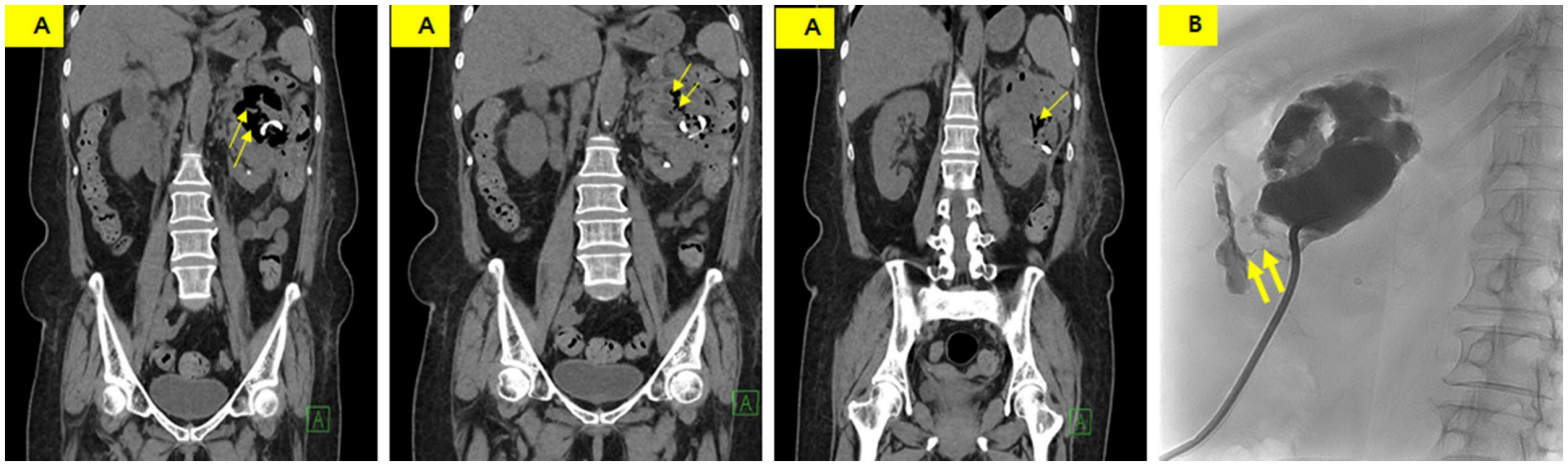
**(A)** Non-enhanced CT showing a slightly decreased abscess size with new intralesional air (thin arrow) and an indistinct boundary between the perinephric abscess and descending colon. **(B)** Tubogram demonstrating an abscess pocket measuring 7 cm in length, with a fistula tract (thick arrow) showing contrast entering the descending colon.

Non-enhanced CT was performed on the 24th day of hospitalization because of poor drainage and a color change in the drainage. CT showed a decreased size of the perinephric abscess but new air bubbles within it and an edematous change in the descending colon (Figure 3A). These findings strongly suggested the formation of a fistula between the abscess and the descending colon. A tubogram via the PCD catheter revealed a fistula between the perinephric abscess and the descending colon (Figure 3B).

**Figure 4:**
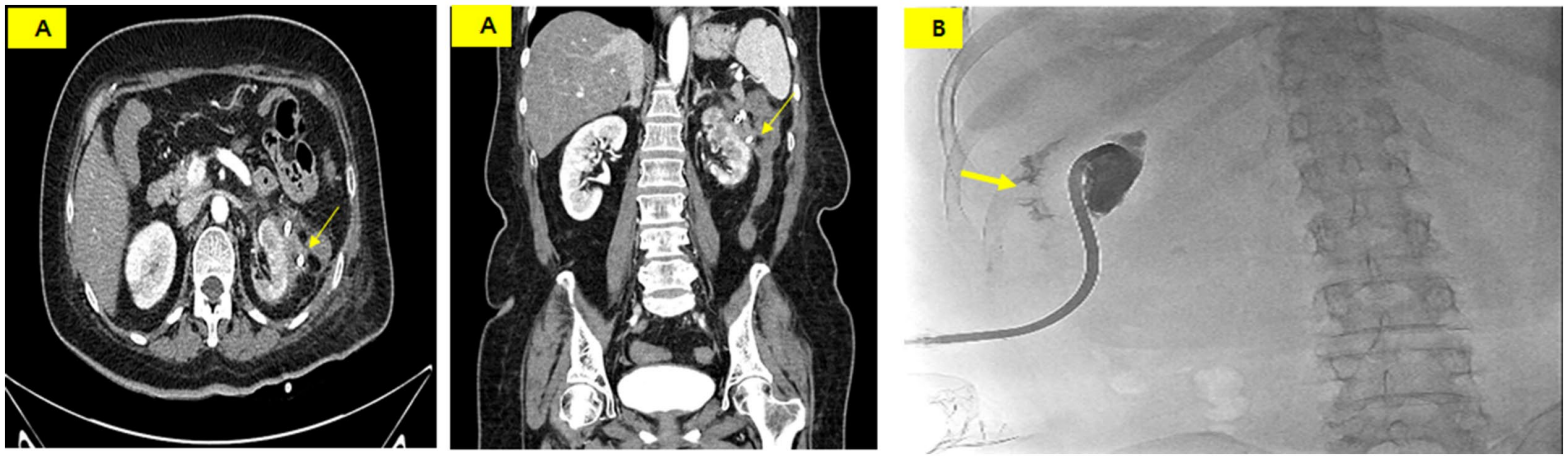
**(A)** CT scan on hospital day 60 showing a marked reduction of the abscess and a suspicious fistula or adhesion between the perinephric abscess and the descending colon (thin arrow). **(B)** Tubogram showing residual contrast leakage into the colon (thick arrow) but near resolution of the fistula tract.

An ileostomy was performed to prevent fecal material from passing into the perinephric abscess on the 26th day of hospitalization. Draining of the fluid culture at this time revealed complicating pathogens, including *Klebsiella pneumoniae*, *Enterococcus faecium*, *Candida albicans*, and *Candida glabrata*. Vancomycin, ceftriaxone, and micafungin were administered intravenously. On the 30th day of hospitalization, the PCD catheter was removed as the infection spread from the abscess to the skin along the catheter tract. The PCD catheter was then reinserted at another site. Subsequently, tube repositioning was performed once every 7 days to maintain the drainage volume. A CECT performed on the 60th day of hospitalization showed that the abscess had nearly disappeared (Figure 4A). A tubogram via the PCD catheter showed contrast leakage into the descending colon but nearly complete disappearance of the previous prominent fistula tract (Figure 4B).

On the 65th day of hospitalization, the PCD catheter was removed as no drainage was observed for 5 consecutive days. The patient was discharged on the 70th day of hospitalization with an ileostomy in place and is currently undergoing outpatient follow-up without any other complications. Ileostomy repair is scheduled to be performed in the Department of Surgery.

## Discussion

We are the first to report a case of idiopathic WS complicated by a perinephric abscess accompanied by a fistula to the descending colon. The primary event appeared to be an unexplained non-traumatic spontaneous renal hemorrhage into the subcapsular space, followed sequentially by perinephric abscess formation due to a secondary bacterial infection, which is presumed to have subsequently led to a fistula to the descending colon. Using a systematic approach, the patient was accurately diagnosed and received appropriate management, resulting in complete recovery without severe complications despite prolonged hospitalization.

The classical triad of WS comprises unilateral flank pain, generalized malaise with hypovolemic shock, and a palpable lumbar mass (Lenk’s triad) (8). However, the presence of all elements of Lenk’s triad is uncommon, occurring in only 20% of all WS cases (9). Flank or abdominal pain is the most common manifestation of WS and is seen in 67% of patients, while hematuria (40%) and hypovolemic shock (27%) may occur in isolation or in concert (9). Other reports stated that flank pain was observed in 92% of patients, gross hematuria in 8%, and microscopic hematuria in 39% (10). A meta-analysis of WS confirmed that 83% of patients presented with acute-onset flank or abdominal pain, 19% had hematuria, and 11% had symptoms and signs of hypovolemic shock (11). The patient denied a history of trauma before the abrupt onset of left flank pain, and we could not find any evidence of trauma on physical examination. Microscopic or gross hematuria, flank mass, and hypovolemic shock were not observed in the patient. Even in cases where the patient complains only of abdominal or flank pain without a history of trauma or other symptoms and signs, WS should be reasonably suspected, and radiologic imaging studies must be conducted.

The main cause of WS is renal neoplasm (57–63%), most frequently angiomyolipoma (AML), followed by renal cell carcinoma (RCC) (1, 4, 10). These neoplasms usually display an increased proclivity for hemorrhage and rupture. Various renal vascular diseases, such as aneurysms or pseudo-aneurysms, arteriovenous malformations or fistulae, and vasculitis syndromes, account for 20–30% of WS cases (4). Rare causes of WS include APN, cystic renal disease, end-stage kidney disease, and coagulation disorders (4, 10–12), while some WS cases are idiopathic (4); however, these may be related to the rupture of small cysts or small extrarenal vessels, infection, and passed calculi (13). Sometimes, the cause may be discovered in surgical specimens (10, 11). Multiphasic CT or MRI is essential for the detection, localization, and characterization of the underlying causes and aids in the planning of optimal management (4). Emekli and Gundogdu (14) proposed that CT characteristics are essential in the diagnosis and management of WS to identify hemodynamically unstable cases in need of immediate intervention. In cases of large-volume hemorrhage where CT or MRI initially cannot identify any contributory causes, a serial CT or MRI follow-up study may be necessary to establish the cause of WS. We could not find any cause of WS despite a follow-up CT study after relieving the hemorrhage volume, and the patient did not take any medication that had a bleeding tendency, such as warfarin and direct oral anticoagulants (DOACs). We excluded renal infection as a cause of WS because the patient did not complain of fever or show symptoms of a lower urinary tract infection, and no compatible findings were found on CECT. Furthermore, urinalysis did not show pyuria, and bacteria did not grow in the urine and blood cultures.

Although the management of WS is conservative in principle, it widely depends on the presence of active bleeding as well as the patient’s hemodynamic status (3, 14). When active bleeding is detected on CECT or MRI, angiography and selective arterial embolization are the preferred options. Urgent surgical intervention, including partial or radical nephrectomy, is sometimes necessary depending on the clinical situation of the patient, such as the presence of neoplasms or refractory bleeding. Conservative treatments usually include early volume expansion with intravenous fluids and transfusion, immediate removal of causal agents, pain control, and antibiotics (15). The patient received conservative management, including intravenous fluid, antibiotics, and pain control, because her vital signs were stable and active bleeding was not detected on CECT. We initially did not suspect that the subcapsular hematoma was infected with bacteria because she did not present with typical infection signs or symptoms, such as fever, and CECT did not show any evidence of infection, although CRP was mildly elevated. In this case, antibiotics were prophylactically administered.

Perinephric abscess is an uncommon clinical entity that can result in significant morbidity and mortality with complications, including sepsis, renal failure, and fistula formation (5). Perinephric abscess with fistula formation to the descending colon is considered a rare and complex clinical condition characterized by abnormal communication between the kidney and colon. Several patient-specific factors may have contributed to the unusual progression from spontaneous renal hemorrhage to secondary infection, abscess formation, and eventual fistulization. The patient had long-standing diabetes mellitus with suboptimal glycemic control (HbA1c 8.5%), which likely impaired local immune responses and increased her susceptibility to bacterial infections. Only one case of fistula formation to the descending colon has been reported (15). Duran et al. (16) presented a case report of a perinephric abscess following a percutaneous CT-guided kidney biopsy fistulized with the descending colon. They asserted that a hematoma was first formed by biopsy and subsequently became infected. In this case, a subcapsular hematoma developed spontaneously and was subsequently infected with bacteria. This persistent inflammation resulted in fistula formation in the colon.

Early diagnosis of perinephric abscess or colon fistula is highly challenging because of the absence of typical clinical manifestations. CT remains an important diagnostic tool that can detect the presence of a fistula. We suspected a perinephric abscess to the colon fistula after observing newly formed air bubbles within the abscess pockets on follow-up CT. A definite diagnosis of fistula formation was established via a catheter tubogram, with a drainage catheter used in this case. We also confirmed the fistula tract using colonoscopy. The mainstay of treatment for perinephric abscess is drainage. Antibiotics are mainly used as an adjunct to percutaneous drainage because they help control sepsis and prevent the spread of infection (17). To prevent fecal material from the colon from entering the abscess pockets through the fistula, an ileostomy was performed. When the drainage amount decreased, timely catheter repositioning or reinsertion was performed to maintain effective drainage, and these efforts ultimately led to nearly complete resolution of a large abscess measuring 11 cm in length.

This case also offers important clinical implications for managing spontaneous renal hemorrhage. Even in patients with idiopathic WS and no overt signs of infection, secondary bacterial contamination of a hematoma may occur in individuals with metabolic risk factors such as long-standing diabetes. Therefore, early repeat imaging, close monitoring of symptom progression, and timely drainage are essential to prevent progression to abscess formation and fistulization.

In conclusion, idiopathic WS may progress to secondary infection, leading to rare but serious complications, including perinephric abscess and fistula formation involving adjacent organs. Prompt imaging reassessment and multidisciplinary management are essential for achieving favorable outcomes.

## Data Availability

The original contributions presented in the study are included in the article/supplementary material, further inquiries can be directed to the corresponding author.
